# Correction: Age-related differences in resting-state, task-related, and structural brain connectivity: graph theoretical analyses and visual search performance

**DOI:** 10.1007/s00429-024-02887-0

**Published:** 2025-01-02

**Authors:** David J. Madden, Jenna L. Merenstein, Hollie A. Mullin, Shivangi Jain, Marc D. Rudolph, Jessica R. Cohen

**Affiliations:** 1 Brain Imaging and Analysis Center, Duke University Medical Center, Box 3918, Durham, NC 27710 USA; 2 Department of Psychiatry and Behavioral Sciences, Duke University Medical Center, Durham, NC 27710 USA; 3 Center for Cognitive Neuroscience, Duke University, Durham, NC 27708 USA; 4 Department of Psychology and Neuroscience, University of North Carolina at Chapel Hill, Chapel Hill, NC 27514 USA; 5 Present Address: Department of Psychology, Pennsylvania State University, University Park, PA 16802 USA; 6 Present Address: AdventHealth Research Institute, Neuroscience Institute, Orlando, FL 32804 USA; 7 Present Address: Department of Internal Medicine, Wake Forest University School of Medicine, Winston-Salem, NC 27101 USA

**Correction: Brain Structure and Function (2024) 229:1533-1559**

10.1007/s00429-024-02807-2

The article “Age-related differences in resting-state, task-related, and structural brain connectivity: graph theoretical analyses and visual search performance” written by David J. Madden, Jenna L. Merenstein, Hollie A. Mullin, Shivangi Jain, Marc D. Rudolph, Jessica R. Cohen was originally published Online First without Open Access. After publication in volume 229, issue 7, page 1533–1599 the author decided to opt for Open Choice and to make the article an Open Access publication. Therefore, the copyright of the article has been changed to © The Author(s) 2024 and the article is forthwith distributed under the terms of the Creative Commons Attribution 4.0 International License, which permits use, sharing, adaptation, distribution and reproduction in any medium or format, as long as you give appropriate credit to the original author(s) and the source, provide a link to the Creative Commons licence, and indicate if changes were made. The images or other third party material in this article are included in the article’s Creative Commons licence, unless indicated otherwise in a credit line to the material. If material is not included in the article’s Creative Commons licence and your intended use is not permitted by statutory regulation or exceeds the permitted use, you will need to obtain permission directly from the copyright holder. To view a copy of this licence, visit http://creativecommons.org/licenses/by/4.0.

Open access funding enabled and organized by Projekt DEAL.

The original article has been corrected.

